# Incidental Finding of an Asymptomatic Perielectrode Cyst After Deep Brain Stimulation for Parkinson’s Disease

**DOI:** 10.7759/cureus.116054

**Published:** 2026-09-10

**Authors:** Stephanie Du Four, Stijn Vandamme, Dimitri Vanhauwaert, Jeroen Van Lerbeirghe, Olivier Van Damme

**Affiliations:** 1 Neurosurgery, Erasmus Brussels University of Applied Sciences and Arts, Brussels, BEL; 2 Neurosurgery, AZ Delta, Roeselare, BEL; 3 Human Structure and Repair, Ghent University, Ghent, BEL

**Keywords:** deep brain stimulation, lead complication, parkinson’s disease, perielectrode cyst, subthalamic nucleus

## Abstract

Implanted intracerebral devices can lead to infection, hemorrhage, or the formation of fluid-filled cysts. Formation of a perielectrode cyst after deep brain stimulation (DBS) has been reported in a limited number of patients. Additionally, diverse causes and clinical presentations have been associated with these cysts. We present a case of perielectrode cyst formation three months after implantation of a subthalamic nucleus DBS (STN-DBS) for Parkinson’s disease (PD). A 59-year-old woman was diagnosed with PD in 2011. In November 2023, the patient underwent staged placement of an STN-DBS without perioperative complications. Immediate postoperative cerebral CT ruled out acute complications. Postoperatively, the patient showed clear clinical improvement, and a substantial reduction in the levodopa dose was achieved (1,500 mg/24 h vs. 250 mg/24 h). There was a significant change in neurocognitive testing three months postoperatively, with a decrease in the Montreal Cognitive Assessment score from 29/30 to 24/30. Follow-up cerebral MRI showed a perielectrode cystic lesion around the left electrode. The patient’s clinical status and electrode impedances remained unchanged during follow-up. Serial imaging demonstrated gradual regression of the cyst. Perielectrode cysts after DBS can occur at different locations and at varying postoperative time points. They may present with diverse clinical manifestations or remain completely asymptomatic. Different putative mechanisms and treatment options have been described; however, the paucity of reported clinical cases makes it difficult to speculate on a primary cause.

## Introduction

Deep brain stimulation (DBS) is an established treatment for various neurological disorders [1]. Although it is generally considered safe and effective, complications such as infection, hardware-related issues, and perielectrode cyst formation can occur.

A perielectrode cyst is a fluid collection surrounding a DBS lead. Compared with other complications, such as perielectrode edema (3.1-40%), cerebral hemorrhage (1-8%), wound infection (3-10%), and postoperative seizures (3.1%), perielectrode cyst formation is rare, with a reported incidence of 0.8% [2-4]. These cysts may present acutely or with delayed onset, with the time to diagnosis ranging from several hours to 59 months postoperatively. Their clinical presentation can vary from asymptomatic to focal neurological deficits or signs of infection [3].

The etiology of perielectrode cysts remains unknown. Different underlying mechanisms have been proposed, including perielectrode edema, microhemorrhage, infection, venous infarction, and inflammatory reaction [3]. Due to the rarity of perielectrode cysts, there is limited literature on their management. There are no standardized recommendations for follow-up imaging or treatment.

We present the case of a 59-year-old woman who developed a perielectrode cyst after DBS surgery for Parkinson’s disease (PD). A postoperative MRI performed because of cognitive decline revealed the presence of a cyst surrounding the left DBS electrode.

## Case presentation

A 59-year-old woman with a 13-year history of PD and an LRRK2 mutation underwent bilateral subthalamic nucleus DBS (STN-DBS) at our clinic. She was selected for DBS because of disabling peak-dose dyskinesias and motor fluctuations. Standard multidisciplinary screening evaluations were performed. On the Movement Disorder Society-sponsored revision of the Unified Parkinson’s Disease Rating Scale Part III, her score was 1 in the ON state and 30 in the OFF state [5]. During the cognitive evaluation, she scored 29/30 on the Montreal Cognitive Assessment (MoCA) [6]. Based on these findings, she was selected for bilateral STN-DBS surgery. Apart from PD, she had no relevant medical history.

The patient underwent uncomplicated bilateral STN-DBS under general anesthesia. At our institution, STN-DBS is performed using an asleep technique with perioperative imaging and without microelectrode recordings. The electrodes did not require repositioning during surgery. Correct electrode placement was verified by fusion of the preoperative imaging with the perioperative O-arm three-dimensional scan. Cartesia directional leads (30 cm; Boston Scientific, Marlborough, MA, USA) were implanted and connected to a Genus implantable pulse generator (Boston Scientific) after imaging confirmation. There was no excessive irrigation of the surgical field with saline or any other solution. The total duration of surgery was 140 minutes.

Immediate postoperative cerebral CT ruled out hemorrhage and other acute lesions. The patient tolerated the surgery well and was discharged home after an uneventful postoperative course. Postoperatively, she showed clear clinical improvement, and a substantial reduction in the levodopa dose was achieved (1,500 mg/24 h vs. 250 mg/24 h).

Three months postoperatively, routine cerebral MRI revealed a cystic structure measuring approximately 2.6 mm surrounding the distal portion of the left STN electrode. The lead coordinates changed significantly (Figure 1, Table 1), but there were no changes in electrode impedances (Table 2, Table 3).

**Figure 1:**
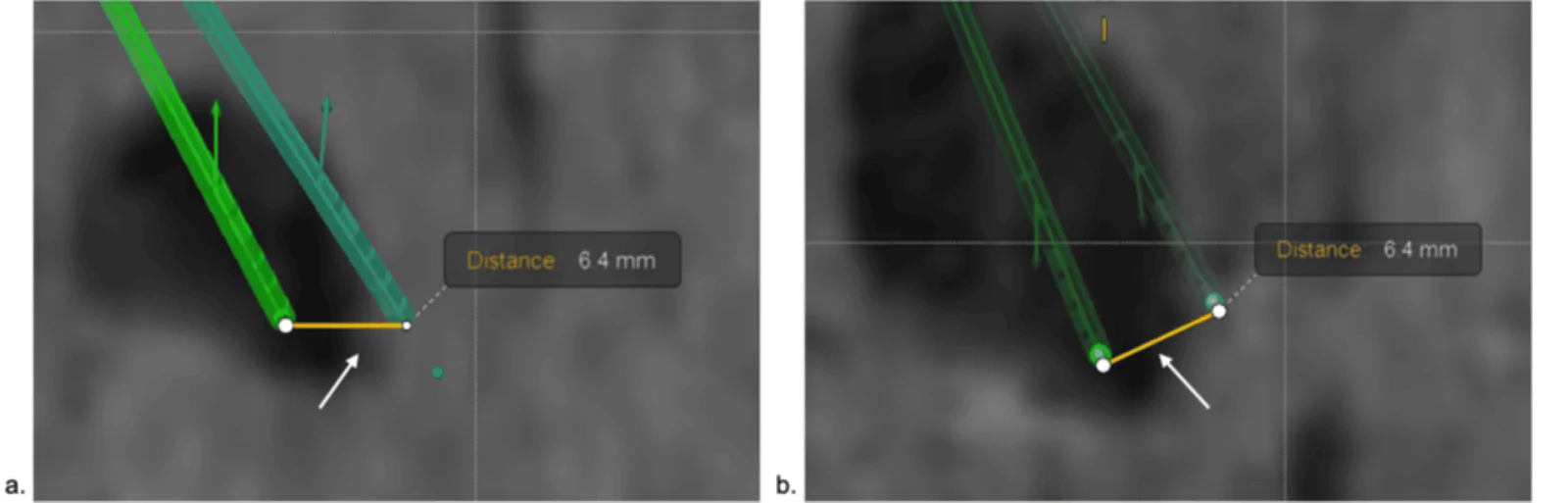
Displacement of the left electrode by the cyst, as determined using the Brainlab software (Brainlab AG, Munich, Germany) on cerebral MRI: (a) axial T1-weighted image and (b) coronal T1-weighted image. The white arrows indicate the displaced left electrode.

**Table 1:** Coordinates of the left DBS lead immediately postoperatively and at the time of cyst diagnosis. AC/PC, anterior commissure/posterior commissure; MC, midpoint of the anterior commissure-posterior commissure line

Postoperative AC/PC coordinates	
Reference point	MC
Target	
Lateral left	9.12 mm
Posterior	2.79 mm
Inferior	6.17 mm
Angulation	
Lateral left	22.15°
Anterior	37.42°
AC/PC coordinates at diagnosis of cyst	
Reference point	MC
Target	
Lateral left	3.33 mm
Posterior	2.72 mm
Inferior	3.41 mm
Angulation	
Lateral left	21.27°
Anterior	33.67°

**Table 2:** Impedances of the left DBS electrode at the time of perielectrode cyst diagnosis. DBS, deep brain stimulation; STN, subthalamic nucleus

Lead type		Impedances (Ω)							
DN-2202		Case	E8	E7	E6	E5	E4	E3	E2
Hemisphere left	E8	1136							
	E7	2842	3554						
	E6	2936	3657	4945					
Target STN	E5	2325	3081	4466	4469				
	E4	3100	3893	5325	5570	4981			
Lead orientation anterior	E3	3725	4502	6072	6015	5576	5864		
	E2	2802	3618	5222	5307	4590	5099	5733	
	E1	1204	2138	3782	3863	3253	3853	4474	3569

**Table 3:** Impedances of the right DBS electrode at the time of perielectrode cyst diagnosis. DBS, deep brain stimulation; STN, subthalamic nucleus

Lead type		Impedances (Ω)							
DN-2202		Case	E8	E7	E6	E5	E4	E3	E2
Hemisphere right	E8	1445							
	E7	3364	4170						
	E6	3419	4232	5707					
Target STN	E5	3547	4348	5805	5902				
	E4	3962	4678	6289	6490	6647			
Lead orientation anterior	E3	3856	4829	6588	6490	6798	6446		
	E2	3370	4386	6164	6210	6178	5869	6144	
	E1	1595	2695	4574	4619	4757	4665	4819	4254

The patient remained asymptomatic, with sustained clinical improvement, and electrode impedances remained stable. Serial imaging at the six- and nine-month follow-up demonstrated gradual regression of the perielectrode cyst (Figure 2). The MoCA score improved to 27/30 at the nine-month follow-up.

**Figure 2:**
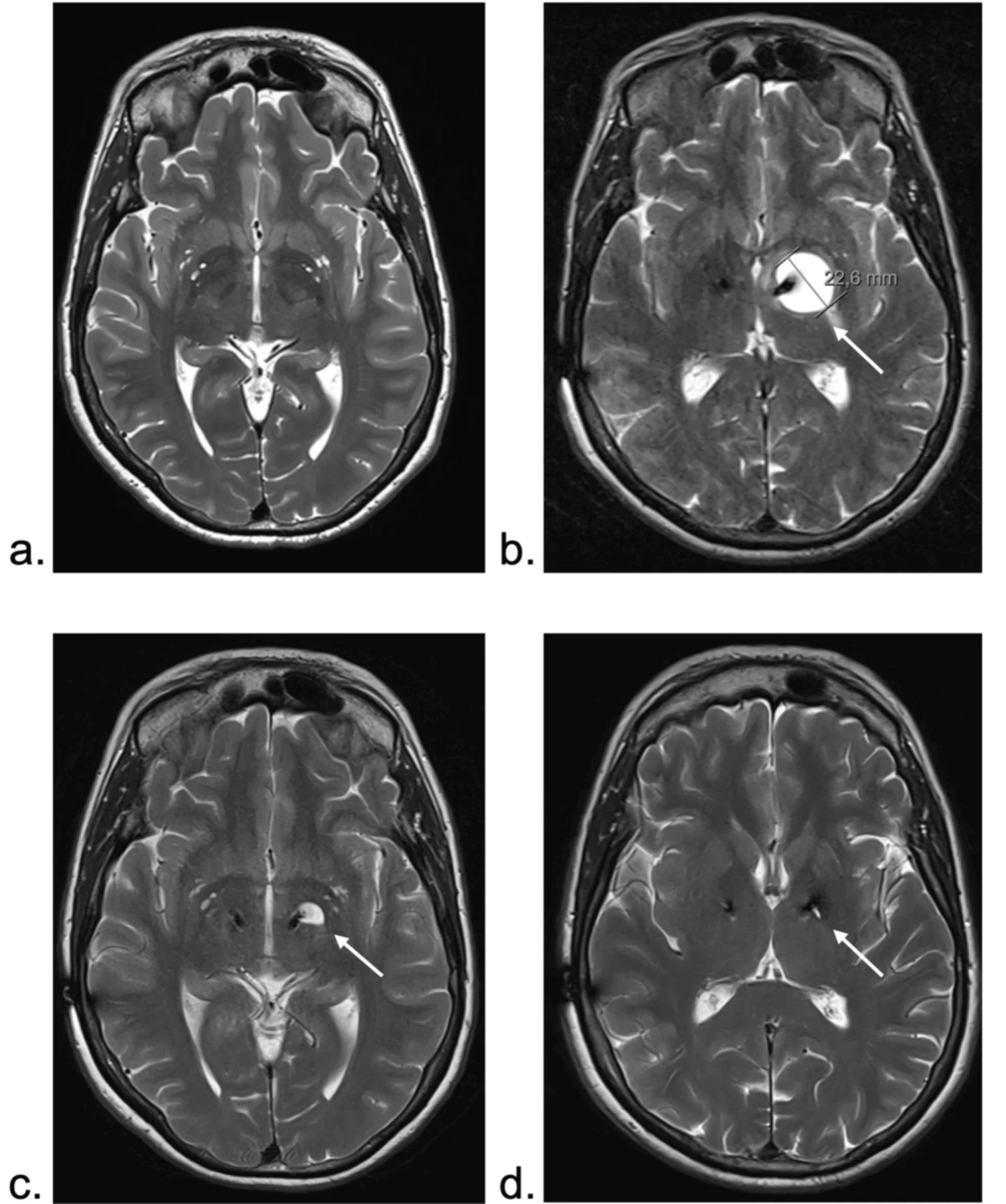
Evolution of the perielectrode cyst on cerebral MRI. (a) Serial T2-weighted MRI images demonstrating the preoperative cerebral MRI. (b) Cerebral MRI at the time of perielectrode cyst diagnosis (white arrow). (c) Six-month follow-up cerebral MRI showing spontaneous involution of the cyst (white arrow). (d) Nine-month follow-up cerebral MRI showing near-complete resolution of the cyst (white arrow).

In the present case, timely recognition of the cyst and a wait-and-scan approach resulted in spontaneous regression of the cyst. Close collaboration among neurosurgeons, neurologists, neuroradiologists, and DBS nurses is crucial in the management of these patients.

## Discussion

Perielectrode cyst formation following DBS surgery is a rare but potentially life-threatening complication. The clinical presentation of perielectrode cysts is variable. Some patients remain asymptomatic, whereas others present with acute-onset or progressive neurological symptoms [6,7]. Depending on the clinical presentation, treatment of perielectrode cysts ranges from a wait-and-scan approach or corticosteroid therapy to surgical drainage and/or lead removal [8-12].

The exact pathophysiology of cyst formation remains unclear. In a retrospective report of complications after DBS placement, only 0.8% of patients developed a perielectrode cyst [2]. Another series reported an incidence of 6.3% [10,11]. In these cases, the authors suggested that the cyst develops as the natural progression of untreated or persistent perielectrode edema. The proposed mechanism involves accumulation of CSF from the subarachnoid space along the lead due to disruption of ependymal integrity [8,10,13,14]. However, perilesional edema is typically present within the first four to 120 days postoperatively and usually resolves within one to two months. In previously reported patients, the mean time to development of symptomatic cysts was 6.2 months [2,10]. Therefore, the relationship between perilesional edema and cyst formation remains uncertain. Other proposed mechanisms include microhemorrhage, mechanical trauma, an inappropriate immune response, and CSF tracking along the electrode [3,11].

In our patient, there were no preexisting Virchow-Robin spaces along the planned trajectory on preoperative cerebral MRI. Additionally, immediate postoperative cerebral CT showed no edema surrounding the electrode.

In our case, the perielectrode cyst was identified during the evaluation of cognitive decline, as reflected by a decreased MoCA score. Although the MoCA score subsequently improved, it remains unclear whether the cognitive decline was related to the perielectrode cyst because there were no signs of perilesional edema and the cyst was unilateral. Only one other case in which cognitive decline led to the diagnosis of a perielectrode cyst has been reported. In that case, bilateral perielectrode edema progressed to bilateral cysts. The patient was treated with corticosteroids, and both the edema and cysts improved after nine months. Similar to our patient, motor benefit from DBS was maintained [15].

Different treatment strategies have been proposed for perielectrode cysts. In symptomatic cases, treatment with corticosteroids has resulted in cyst regression. In most reported cases, the hardware was removed because of progressive neurological deficits or to rule out infection [3]. Because our patient experienced sustained motor benefit with a normally functioning DBS system and stable electrode impedances, follow-up with serial imaging was chosen. Similarly, spontaneous regression of perielectrode cysts has been reported in asymptomatic patients managed conservatively [12,13]. Given the considerable variability in the clinical and radiological course of perielectrode cysts, close follow-up with serial imaging to monitor lead coordinates and careful monitoring of electrode impedances are essential when adopting a conservative wait-and-scan approach.

## Conclusions

Perielectrode cyst formation is a rare and potentially dangerous complication following DBS surgery. Because routine imaging is not commonly performed, perielectrode cysts are likely underdiagnosed and underreported. Symptoms are most commonly progressive, although sudden onset has also been reported. Although most reported cases have been treated with hardware removal, observation with serial imaging is a possible management strategy because spontaneous cyst regression has been described. Close patient follow-up and multidisciplinary collaboration are essential.
